# Overt disseminated intravascular coagulation and antithrombin III predict bleeding and in-hospital mortality in patients undergoing extracorporeal membrane oxygenation

**DOI:** 10.3389/fmed.2024.1335826

**Published:** 2024-04-22

**Authors:** Tae Wan Kim, Ryoung-Eun Ko, Ki Hong Choi, Chi Ryang Chung, Yang Hyun Cho, Jeong Hoon Yang

**Affiliations:** ^1^Department of Pulmonary and Critical Care Medicine, Chung-Ang University Hospital, Chung-Ang University College of Medicine, Seoul, Republic of Korea; ^2^Department of Critical Care Medicine, Samsung Medical Center, Sungkyunkwan University School of Medicine, Seoul, Republic of Korea; ^3^Division of Cardiology, Department of Medicine, Heart Vascular Stroke Institute, Samsung Medical Center, Sungkyunkwan University School of Medicine, Seoul, Republic of Korea; ^4^Department of Thoracic and Cardiovascular Surgery, Samsung Medical Center, Sungkyunkwan University School of Medicine, Seoul, Republic of Korea

**Keywords:** disseminated intravascular coagulation, extracorporeal membrane oxygenation, antithrombin III, bleeding, thrombosis

## Abstract

**Background:**

Limited data are available on the relationship of disseminated intravascular coagulation (DIC) with mortality in patients receiving extracorporeal membrane oxygenation (ECMO). Thus, we investigated the association of DIC score and antithrombin (AT) III with clinical outcomes in patients undergoing ECMO.

**Methods:**

We analyzed 703 patients who underwent ECMO between January 2014 and May 2022 at Samsung Medical Center. The DIC score was calculated using laboratory findings within 24 h of the ECMO initiation, and ≥ 5 was defined as overt DIC. In addition, the AT III level was measured to identify the correlation with the DIC score.

**Results:**

Among the study patients, 169 (24.0%) were diagnosed with overt DIC (DIC group) during early maintenance therapy. In-hospital mortality was significantly higher in the DIC group than in the non-DIC group (55.0% vs. 36.5%, *p* < 0.001). Bleeding events were significantly higher in the group of patients with a DIC score of 7 or 8 than in the other group (20.8% vs. 8.4%, *p* = 0.038). DIC score negatively correlated with AT III level (*r* = −0.417, *p* < 0.001). The predictive performance of AT III for overt DIC had statistical significance with a c-static of 0.81 (95% confidence interval (CI), 0.77–0.84, *p* < 0.001).

**Conclusion:**

Overt DIC was associated with higher in-hospital mortality and a tendency to bleed in ECMO patients. Furthermore, AT III plasma levels can easily predict overt DIC in patients undergoing ECMO. These findings suggest that monitoring AT III plasma levels may be important in the management of ECMO.

## Introduction

Disseminated intravascular coagulation (DIC) is an acquired syndrome characterized by systemic intravascular activation of coagulation that results in intravascular fibrin formation (1) and is associated with severe infection, major trauma, and immunological and hematological disorders (2). DIC may eventually lead to the deterioration of oxygen supply to tissues due to the thrombotic occlusion of blood vessels (1) and is a major cause of death among critically ill patients (3).

In general, extracorporeal circulation induces systemic inflammation and coagulation activation due to the exposure of the patient’s blood to non-endothelialized surfaces of the extracorporeal membrane oxygenation (ECMO) circuit (4). In previous studies, the incidence of DIC was reported to be very high, exceeding 40% in patients supported with ECMO (5); however, the prognostic role of DIC in the ECMO setting was inconclusive because previous observational studies were conducted on specifically targeted populations, such as septic shock or cardiac surgery patients, and the number of patients was very limited (5, 6). In addition, antithrombin (AT) deficiency might be caused by activated coagulation and long-term anticoagulation, impaired synthesis, and DIC (7), particularly acquired AT deficiency, a common occurrence in patients supported with ECMO (8). However, the clinical importance of monitoring AT III as a biomarker has not been fully elucidated because low AT III supplementation (93.0–123.0%) did not improve clinical outcomes in patients with acute respiratory failure undergoing venovenous ECMO, although it may help reduce the imbalance between pro-coagulant and anticoagulant factors (9).

Therefore, we investigated the association of DIC score and AT III with clinical outcomes and evaluated the predictive value of AT III for overt DIC in patients receiving either venovenous or venoarterial ECMO.

## Methods

### Study population

**Figure 1:**
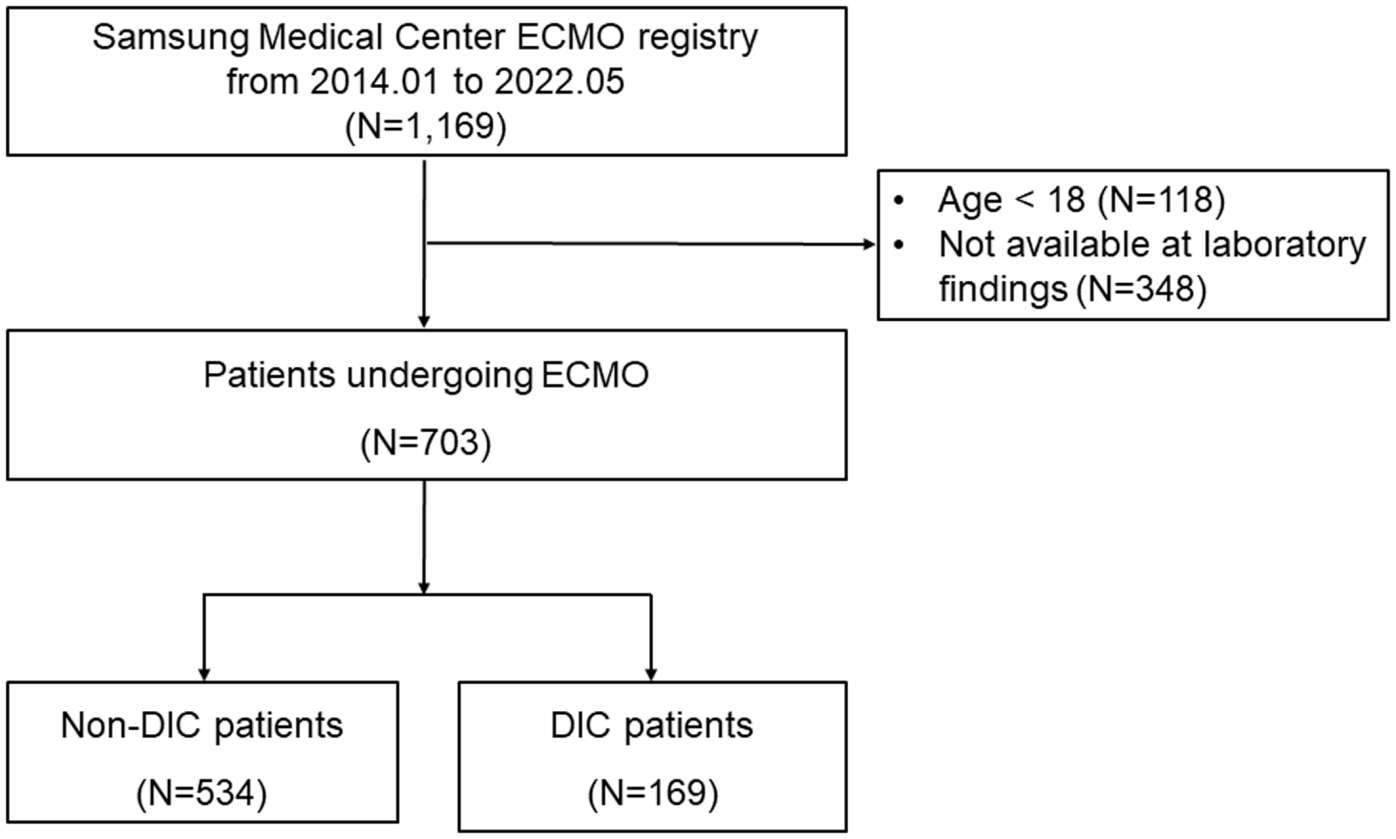
Study flowchart. DIC, disseminated intravascular coagulation; ECMO, extracorporeal membrane oxygenation.

All consecutive patients who received ECMO between January 2014 and May 2022 at Samsung Medical Center were eligible for this study. Among 1,169 patients who underwent ECMO, 466 patients who were < 18 years of age or had missing data needed to calculate the DIC score were excluded (Figure 1). The Institutional Review Board of Samsung Medical Center approved this study (IRB. No. 2022-12-147) and waived the requirement for informed consent due to the retrospective, observational nature of the study. Patient information was anonymized and de-identified before analysis.

### Definitions and outcome

The DIC score was routinely checked daily based on platelet count, prothrombin time, fibrinogen, and D-dimer titer. We calculated DIC scores with values closest to ECMO start time among laboratory findings within 24 h before and after ECMO start time. A DIC score ranged from 0 to 8 points, and a score of 5 or higher was defined as overt DIC according to the International Society on Hemostasis and Thrombosis (Supplementary Table S1) (10). AT III plasma levels were measured in the AT activity concentration of the plasma sample using the STA-R Max (Stago, France) equipment. This is a chromogenic assay-based test that measures the absorbance at 405 nm after adding bovine thrombin to a patient’s platelet-poor plasma. The normal range of the AT III assay in our laboratory was 83–123%. AT III plasma levels were measured within 3 days of ECMO initiation because they are measured every Tuesday and Thursday based on our institutional protocol to identify the status of coagulopathy. The AT III plasma level measurement and the DIC score calculation date are taken the same day; if not, the time difference did not exceed 72 h. Severe AT III deficiency was defined as an AT III plasma level < 50% (11).

The primary outcome was in-hospital mortality. Secondary outcomes were bleeding, thrombosis, and successful ECMO weaning. Bleeding was defined according to the Bleeding Academic Research Consortium (BARC) as major bleeding or minor bleeding (12). Thrombosis was defined as one or more of the following conditions: cerebral infarction, systemic embolization such as the spleen, renal, and thrombus in an artery or vein, and/or major component (membrane, blood pump, and entire circuit) of the device occurring within 7 days of ECMO initiation. Successful ECMO weaning was defined as being free from ECMO and alive for 48 h after decannulation (13, 14).

### Statistical analysis

Continuous variables were presented as mean ± standard deviation or median (25th to 75th percentiles), and differences were compared using the Student’s *t*-test or Wilcoxon rank-sum test when applicable. Categorical variables were expressed as numbers and percentages and analyzed using the chi-square tests or Fisher’s exact tests where applicable. Variables with a *p*-value of <0.05 on univariable analyses and clinically relevant variables were included in the multiple logistic regression model. The odds ratio (OR) with a 95% confidence interval (CI) was reported for each variable. To evaluate the continuous effects of DIC scores and AT III on the outcomes, we used a restricted cubic spline curve with three knots and calculated *p*-values for the interaction between them. The area under the receiver operating characteristic curve (AUROC) was presented to demonstrate the predictive performance of AT III for overt DIC. Statistical analyses were performed using R Statistical Software (Version 3.2.5; R Foundation for Statistical Computing, Vienna, Austria). All tests were two-tailed, and a *p*-value of < 0.05 was considered statistically significant.

## Results

### Clinical characteristics

During the study period, 703 patients were enrolled in this analysis (venoarterial ECMO was performed in 537 patients and venovenous ECMO in 166 patients). Among patients, 169 (24.0%) were diagnosed with overt DIC (DIC group) at the early maintenance phase of ECMO, and 534 were not diagnosed with overt DIC (non-DIC group). Patient characteristics are shown in Table 1, and comorbidities were not significantly different between the two groups. In the laboratory findings at ECMO initiation, platelet (78 × 10^3^/μL, interquartile range (IQR) 49.0–143.0 × 10^3^/μL vs. 193 × 10^3^/μL, IQR 134.0–254.8 × 10^3^/μL; *p* < 0.001), fibrinogen (224 mg/dL, IQR 129.0–335.0 mg/dL vs. 318.5 mg/dL, IQR 224.8–436.0 mg/dL; *p* < 0.001), D-dimer (10.8 μg/mL, IQR 5.5–20.5 μg/mL vs. 2.9 μg/mL, IQR 1.5–8.6 μg/mL; *p* < 0.001), the components required to calculate DIC score, were significantly different between the DIC and non-DIC groups (all *p* < 0.001) and AT III plasma levels (46%, IQR 36.0–56.5% vs. 69%, IQR 55.0–79.0%; *p* < 0.001) were significantly lower in the DIC group.

**Table 1:** Baseline characteristics.

	DIC (*n* = 169)	Non-DIC (*n* = 534)	*p*-value
Age, years	58.4 ± 15.4	57.7 ± 15.1	0.592
Sex, male	115 (68.1)	373 (69.9)	0.728
Body mass index, kg/m^2^	24.1 ± 4.1	24.4 ± 6.4	0.458
Current smoker	31 (18.3)	101 (18.9)	0.898
Comorbidities			
Hypertension	69 (40.8)	229 (42.9)	0.703
Diabetes mellitus	61 (36.1)	192 (34.5)	0.779
Chronic kidney disease^a^	21 (12.4)	56 (10.5)	0.574
Cerebrovascular disease	15 (8.9)	40 (7.5)	0.674
Indications for ECMO			0.091
Cardiac arrest	78 (46.2)	213 (39.9)	
Cardiogenic shock	84 (49.7)	310 (58.1)	
Respiratory failure	3 (1.8)	7 (1.3)	
Others	4 (2.4)	4 (0.8)	
ECMO type			0.479
Venoarterial	133 (78.7)	404 (75.7)	
Venovenous	36 (21.3)	130 (24.3)	
Laboratory findings			
Hemoglobin, g/dL	10.5 (9.1–12.4)	11.5 (9.9–13.5)	<0.001
Platelet, ×10^3^/μL	78.0 (49.0–143.0)	193.0 (134.0–254.8)	<0.001
Total bilirubin, mg/dL	1.3 (0.7–2.7)	0.8 (0.5–1.4)	<0.001
Blood urea nitrogen, mg/dL	26.1 (15.2–42.7)	20.9 (15.3–32.6)	0.027
Creatinine, mg/dL	1.4 (0.9–2.0)	1.1 (0.8–1.6)	<0.001
INR	2.1 (1.7–3.0)	1.2 (1.1–1.4)	<0.001
Fibrinogen, mg/dL	224.0 (129.0–335.0)	318.5 (224.8–436.0)	<0.001
D-dimer, μg/mL	10.8 (5.5–20.5)	2.9 (1.5–8.6)	<0.001
Antithrombin III activity, %	46.0 (36.0–56.5)	69.0 (55.0–79.0)	<0.001
Plasma hemoglobin	23.0 (14.5–40.5)	18.0 (12.0–27.0)	0.005
Troponin I, ng/mL	0.2 (0.0–3.1)	0.4 (0.1–3.1)	0.482
NT-pro BNP, pg/mL	7897.0 (3073.0–33702.0)	2265.0 (383.0–9506.0)	0.005
C-reactive protein, mg/dL	6.5 (1.4–14.1)	1.7 (0.2–8.2)	<0.001

Values are the mean ± standard deviation, median (interquartile range), or n (%).

a Chronic kidney disease is defined as Cr > 2.0 mg/dL or a history of kidney transplantation or maintaining renal replacement therapy.

DIC disseminated intravascular coagulation, ECMO extracorporeal membrane oxygenation, INR international normalized ratio, NT pro-BNP N-terminal pro-brain natriuretic peptide.

### In-hospital management and clinical outcomes

**Figure 2:**
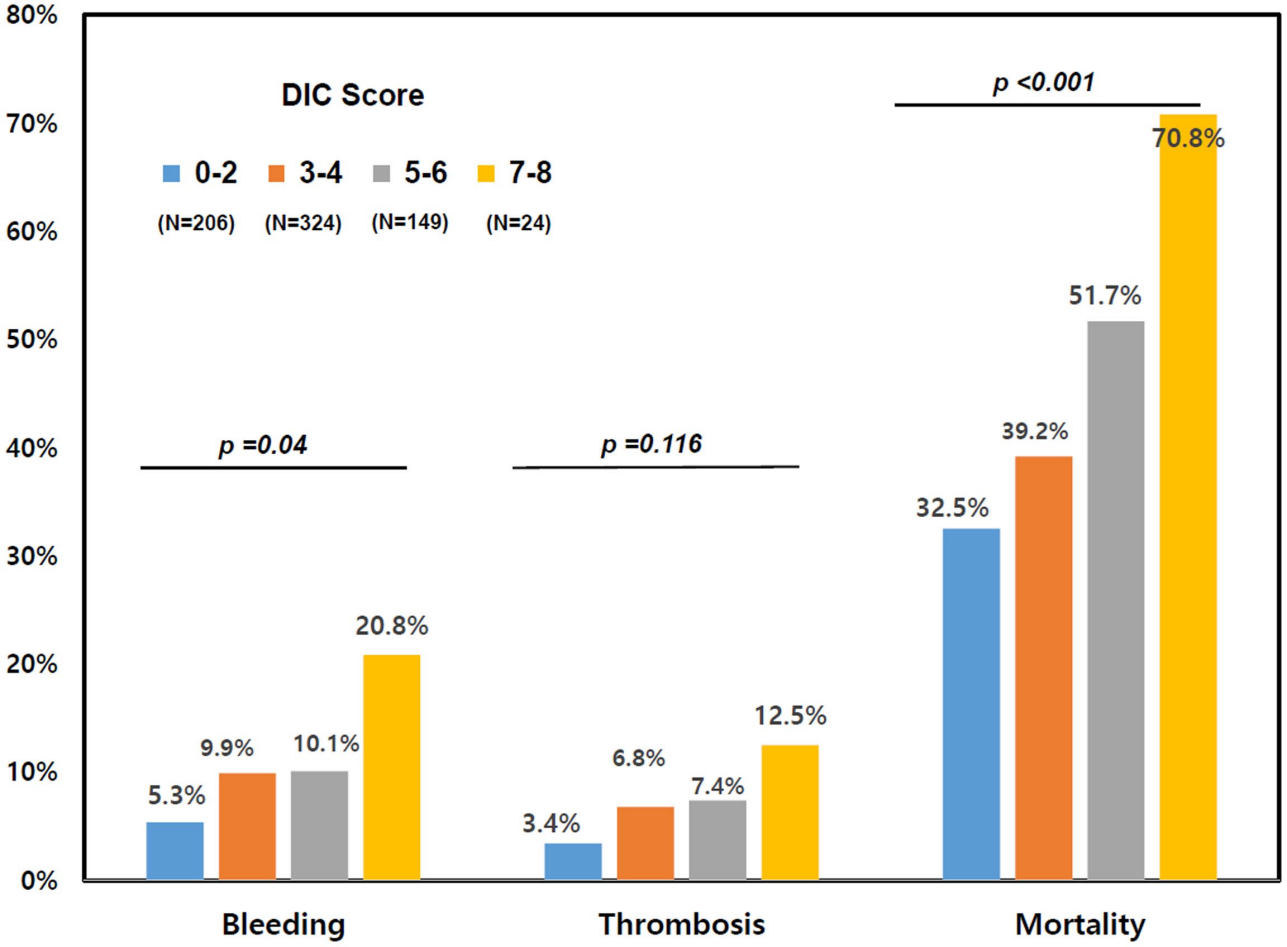
Clinical outcomes based on the DIC score. DIC, disseminated intravascular coagulation.

**Figure 3:**
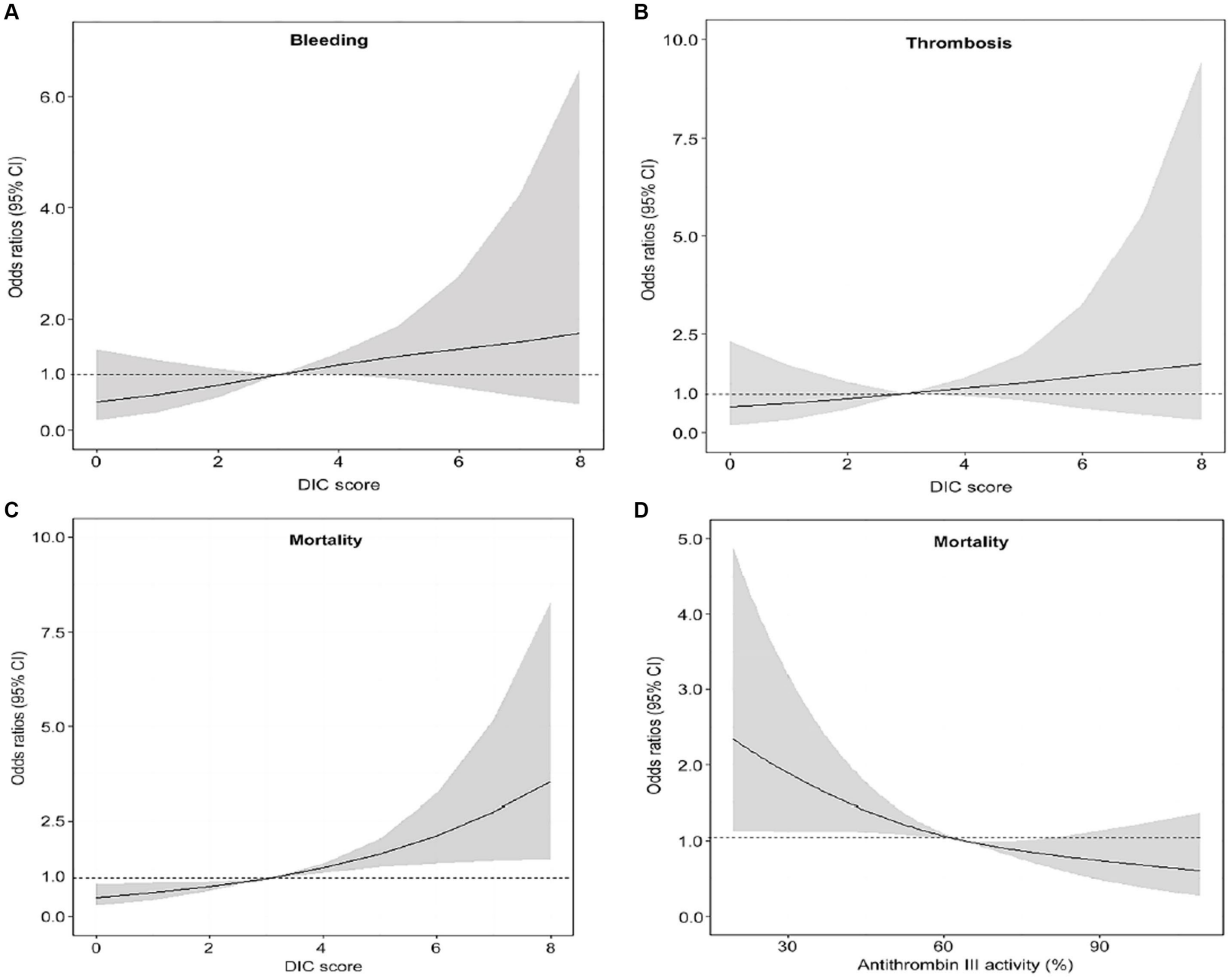
Restricted cubic spline plot of the relationships between DIC score or AT III level and clinical outcomes. **(A)** The association between DIC score and bleeding. **(B)** The association between DIC score and thrombosis. **(C)** The association between the DIC score and mortality. **(D)** The association between AT III and mortality. *AT* antithrombin, *DIC* disseminated intravascular coagulation.

Patients in the DIC group were more likely to receive vasopressors, mechanical ventilation, and continuous renal replacement therapy (CRRT) than subjects in the non-DIC group (Table 2). In-hospital mortality was significantly higher in the DIC group (55.0% vs. 36.5%; *p* < 0.001), and successful ECMO weaning was statistically significantly lower in the DIC group in the total patients and in the venoarterial ECMO patients (56.2% vs. 76.2%; *p* < 0.001 and 57.1% vs. 78.7%; *p* < 0.001, respectively) than in the non-DIC group; however, a significant difference was not observed in venovenous ECMO patients (52.8% vs. 69.5%; *p* = 0.094). In the clinical outcomes, thrombosis (8.3% vs. 5.4%; *p* = 0.244), bleeding (11.8% vs. 7.9%; *p* = 0.153), and ECMO duration (4 days, range 2–9 days 151 vs. 5 days, range 2–10 days; *p* = 0.220) were not significantly different between the two groups. When the DIC score was 7 or 8, the mortality and frequency of bleeding statistically significantly increased (20.8% vs. 8.4%, *p* = 0.038) but without statistical difference in thrombosis (Figure 2). Figure 3 summarizes the continuous ORs for clinical outcomes based on the DIC score and AT III level. The risk of bleeding, thrombosis, and in-hospital mortality tended to increase with increasing DIC score; however, only bleeding and in-hospital mortality were statistically significant (*p* = 0.019 and *p* < 0.001, respectively; Figures 3A–C).

**Table 2:** In-hospital management and clinical outcomes.

	DIC (*n* = 169)	Non-DIC (*n* = 534)	*p*-value
In-hospital management			
Vasopressor	162 (95.9)	461 (87.2)	0.002
Mechanical ventilation	144 (85.2)	418 (78.4)	0.019
CRRT	106 (62.7)	168 (31.7)	<0.001
Intra-aortic balloon pump	4 (2.4)	13 (2.5)	>0.99
Clinical outcomes			
Thrombosis	14 (8.3)	29 (5.4)	0.244
Cerebral infarction	8 (4.7)	13 (2.3)	0.204
Systemic embolization	4 (2.4)	6 (1.1)	0.414
Membrane thrombus	4 (2.4)	13 (2.4)	0.954
Bleeding	20 (11.8)	42 (7.9)	0.153
BARC major	6 (3.6)	12 (2.3)	0.512
BARC minor	14 (8.3)	30 (5.6)	0.287
ECMO duration, days	4 (2–9)	5 (2–10)	0.220
Successful ECMO weaning	95 (56.2)	407 (76.2)	<0.001
Venoarterial	76 (57.1)	318 (78.7)	<0.001
Venovenous	19 (52.8)	89 (69.5)	0.094
In-hospital mortality	93 (55.0)	195 (36.5)	<0.001

Values are the median (interquartile range), or n (%).

DIC disseminated intravascular coagulation, CRRT continuous renal replacement therapy, BARC Bleeding Academic Research Consortium, ECMO extracorporeal membrane oxygenation.

In subgroup analysis, in-hospital mortality was also higher in patients with DIC than subjects with non-DIC, regardless of ECMO type or extracorporeal CPR. In addition, the incidence of thrombotic events was higher in patients with DIC undergoing extracorporeal CPR, although there was no difference between the DIC and non-DIC groups in the overall population (Supplementary Table S2).

### The association of AT III with DIC score and its prognostic role

The OR for mortality decreased as AT III plasma levels increased (*p* = 0.001; Figure 3D). The frequency of each clinical outcome was analyzed based on the severe AT III deficiency (Supplementary Figure S1). Bleeding and thrombosis did not differ, but mortality was significantly higher in the severe AT III deficiency group (49.2% vs. 34.3%; *p* = 0.004). The performance of AT III for the prediction of overt DIC is shown in Supplementary Figure S2; the AUROC curve was 0.81 (95% CI, 0.772–0.844, *p* < 0.001), and the cutoff value was 52.0% with 69.9% sensitivity and 80.7% specificity.

### Factors associated with in-hospital mortality

For the evaluation of clinical factors associated with in-hospital mortality, a univariable analysis was performed using the 12 clinical characteristics of ECMO patients (Table 3). Age, hypertension, chronic kidney disease, extracorporeal cardiopulmonary resuscitation, CRRT, DIC score, BARC major bleeding, and gastrointestinal bleeding were associated with mortality. In a multivariable analysis, age, concomitant cardiac arrest, acute kidney injury requiring renal replacement therapy, DIC score, and gastrointestinal bleeding were associated with an increased risk of mortality.

**Table 3:** Factors associated with in-hospital mortality.

	OR (95% CI)	*p*-value	Adjusted OR^a^ (95% CI)	*p*-value
Age, years	1.02 (1.01–1.03)	<0.001	1.02 (1.01–1.03)	0.002
Sex, male	1.12 (0.81–1.56)	0.497	1.19 (0.82–1.74)	0.367
Smoking	0.73 (0.49–1.08)	0.118	0.77 (0.49–1.20)	0.248
Hypertension	1.58 (1.16–2.14)	0.003	1.37 (0.95–1.98)	0.095
Diabetes mellitus	1.06 (0.77–1.46)	0.714	0.74 (0.51–1.08)	0.114
Chronic kidney disease^b^	2.36 (1.45–3.82)	0.001	1.42 (0.82–2.47)	0.214
ECPR	1.73 (1.27–2.35)	<0.001	1.70 (1.21–2.38)	0.002
CRRT	3.76 (2.73–5.18)	<0.001	2.96 (2.07–4.23)	<0.001
DIC score	1.28 (1.16–1.40)	<0.001	1.16 (1.05–1.29)	0.005
ECMO complications				
BARC major bleeding	2.96 (1.10–7.99)	0.032	2.34 (0.81–6.76)	0.117
Gastrointestinal bleeding	2.51 (1.39–4.55)	0.002	2.29 (1.21–4.35)	0.011
Ischemic stroke	0.71 (0.28–1.79)	0.472	0.51 (0.19–1.39)	0.188

a Adjusted covariates were age, sex, smoking, hypertension, diabetes mellitus, chronic kidney disease, ECPR, renal replacement therapy, DIC score, BARC major bleeding, gastrointestinal bleeding, and ischemic stroke.

b Chronic kidney disease is defined as Cr > 2.0 mg/dL or a history of kidney transplantation or maintaining renal replacement therapy.

DIC disseminated intravascular coagulation, BARC Bleeding Academic Research Consortium, CI confidence interval, ECMO extracorporeal membrane oxygenation, ECPR extracorporeal cardiopulmonary resuscitation, CRRT continuous renal replacement therapy, OR odds ratio.

## Discussion

Our study aimed to investigate the association between overt DIC and clinical outcomes and to determine whether AT III plasma levels could predict overt DIC in ECMO patients using data from a single-center ECMO registry. Our findings revealed that overt DIC (DIC score ≥ 5) was associated with higher in-hospital mortality but not with bleeding or thrombosis, while a DIC score of 7 or 8 was associated with bleeding complications. Moreover, patients with severe AT III deficiency had higher in-hospital mortality, and AT III plasma levels demonstrated good predictive performance for overt DIC in ECMO patients.

DIC is a frequent result of inflammatory states in critically ill patients, and the incidence in patients undergoing ECMO has been reported to range from 41 to 50% (2, 5, 15). The initiation of ECMO is associated with an immediate and complex inflammatory reaction, similar to that observed in systemic inflammatory response syndrome (6). In addition, when the patient’s blood is exposed to the artificial surface of the ECMO circuit, the coagulation–fibrinolysis system and the inflammatory response are activated. These reactions are closely linked through networks of both humoral and cellular components, which can lead to DIC (4). The association of pre-ECMO DIC score with hospital mortality in patients undergoing ECMO has been evaluated in several studies, and the pre-ECMO DIC score was associated with 90-day mortality (16) and a significant risk factor for hospital mortality in patients with septic shock (5). Furthermore, Wang et al. conducted a retrospective study to evaluate the association between DIC score and mortality in 222 patients undergoing venoarterial ECMO after cardiac surgery (6), and 162 (73%) developed DIC within the first day of ECMO initiation. In addition, patients with DIC had higher in-hospital mortality compared with patients with non-DIC. Thus, the authors suggested that an early DIC score might be an important prognostic factor associated with mortality in patients undergoing ECMO after cardiac surgery. However, the association between DIC or coagulopathy and clinical outcomes in patients undergoing ECMO has not been fully elucidated because the focus in previous studies was on septic shock, cardiac arrest, and post-cardiac surgery, and the number of enrolled populations was relatively small. Therefore, the association between DIC score and clinical outcomes was investigated in the present study using a large-scale, dedicated ECMO registry. The results showed the DIC score was a prognostic factor for in-hospital mortality in patients undergoing ECMO due to either cardiac or respiratory failure. In addition, a DIC score of 7 or 8 was associated with a high risk of bleeding complications in the study population, and overt DIC was associated with a high risk of thrombotic complications in cardiac arrest patients who underwent extracorporeal CPR. However, BARC major bleeding and thrombosis represented by ischemic stroke were not a predictor of mortality in the multivariate analysis. This suggests that DIC is not simply an indicator of bleeding and thrombosis, but an indicator that reflects the overall condition of the shock. Indeed, in the DIC group, there were higher frequencies of patients requiring vasopressors, mechanical ventilation, and CRRT, indicating a higher severity of shock. It can be speculated that this contributes to the higher mortality in the DIC group. Additionally, recent advancements in ECMO technology, such as hollow-fiber polymethylpentene oxygenators (17), biopassive surfaces of circuits (18), and enhanced pump and cannula designs (19), are likely to have an impact on the decreasing frequency of bleeding and thrombosis.

Anticoagulation management when undergoing ECMO is generally based on a continuous infusion of unfractionated heparin (20, 21), which is strictly dependent on AT activity in plasma (22, 23). AT inhibits thrombin and activated coagulation factor X; however, the level is not usually measured when diagnosing DIC. In studies in which the comparison of activated clotting time and anti-factor Xa level was evaluated for anticoagulation management in patients undergoing ECMO support, the anti-factor Xa assay was shown to be a more suitable test for anticoagulation monitoring than activated clotting time. However, studies on the association of anti-factor X with DIC are very limited, and further studies are needed (24–26). Recently, Wada et al. analyzed 3,008 DIC patients with severe AT deficiency and found the 28-day survival rate was significantly lower in patients with severe AT deficiency than in subjects without (11). Similarly, mortality was significantly higher in patients with severe AT III deficiency in the present study, and the incidence of bleeding and thrombosis was higher in patients with severe AT III deficiency but without statistical significance. Furthermore, AT III is correlated with DIC and might be a helpful indicator for predicting DIC in patients undergoing ECMO. This finding should be further validated in future studies. In previous studies, the effect of AT supplementation to correct coagulopathy in adult patients requiring ECMO was evaluated; AT supplementation may not reduce heparin requirements, and some subjects do not experience improved heparin responsiveness (9, 27). However, these previous studies were only conducted in patients with acute respiratory failure who underwent venovenous ECMO; thus, future trials are necessary to confirm the effect of AT III supplementation on clinical outcomes in patients with circulatory failure who underwent venoarterial ECMO.

Although the results of the present study provide additional information on the association of DIC and AT III with mortality in patients undergoing ECMO, several limitations should be noted. First, this was a non-randomized cohort study. Therefore, confounding factors and selection bias might have affected the results. Second, because AT III plasma levels are measured twice a week during ECMO maintenance at our institution, the relationship between DIC score and AT III plasma levels should be interpreted with caution because the time difference between DIC score and AT III measurement is minimal. Third, changes in DIC scores during ECMO support were not analyzed. Sequential changes would be more useful data than a single measurement of the DIC score at ECMO initiation. Fourth, findings from the subgroup analysis may not be conclusive due to the underpowered sample size. Finally, due to the lack of data collection on heparin doses as anticoagulants in ECMO patients, the association between AT III and heparin doses is unknown, which is a limitation of this retrospective study.

## Conclusion

In conclusion, overt DIC was associated with a higher in-hospital mortality and a tendency to bleed in patients undergoing ECMO. Additionally, AT III plasma levels can easily predict overt DIC in these patients. These findings suggest that monitoring the DIC score and AT III plasma levels may be important in the management of patients undergoing ECMO.

## Data availability statement

The original contributions presented in the study are included in the article/Supplementary material, further inquiries can be directed to the corresponding authors.

## Ethics statement

The Institutional Review Board of Samsung Medical Center approved this study (approval number: 2022-12-147) and waived the requirement for informed consent due to the retrospective, observational nature of the study. Patient information was anonymized and de-identified before analysis. All procedures were performed in accordance with the ethical standards of the local ethics committee on human experimentation and with the Helsinki declaration of 1975.

## Author contributions

TK: Conceptualization, Data curation, Formal analysis, Investigation, Writing – original draft, Writing – review & editing. R-EK: Conceptualization, Investigation, Methodology, Supervision, Writing – review & editing. KC: Data curation, Investigation, Writing – review & editing. CC: Conceptualization, Investigation, Writing – review & editing, Data curation. YC: Conceptualization, Data curation, Investigation, Writing – review & editing, Methodology. JY: Conceptualization, Formal analysis, Investigation, Methodology, Supervision, Writing – review & editing.
