# Supplementary material for: Overt disseminated intravascular coagulation and antithrombin III predict bleeding and in-hospital mortality in patients undergoing extracorporeal membrane oxygenation
Source: Front Med (Lausanne). 2024 Apr 22;11:1335826. doi: 10.3389/fmed.2024.1335826 (PMC11075479; doi:10.3389/fmed.2024.1335826)
Supplement: Supplementary file 1 [file Data_Sheet_1.docx]

**Supplementary file**

**Overt Disseminated Intravascular Coagulation and Antithrombin III Predict Bleeding and In-Hospital Mortality in Patients Undergoing Extracorporeal Membrane Oxygenation**

Tae Wan Kim, MD^1^; Ryoung-Eun Ko, MD, PhD^2^; Ki Hong Choi, MD^3^; Chi Ryang Chung, MD, PhD^2^; Yang Hyun Cho, MD, PhD^4*^; Jeong Hoon Yang, MD, PhD^2,3*^

^1^Department of Pulmonary and Critical Care Medicine, Chung-Ang University Hospital, Chung-Ang University College of Medicine, Seoul, Korea.

^2^Department of Critical Care Medicine, Samsung Medical Center, Sungkyunkwan University School of Medicine, Seoul, Republic of Korea

^3^Division of Cardiology, Department of Medicine, Heart Vascular Stroke Institute, Samsung Medical Center, Sungkyunkwan University School of Medicine, Seoul, Republic of Korea

^4^Department of Thoracic and Cardiovascular Surgery, Samsung Medical Center, Sungkyunkwan University School of Medicine, Seoul, Republic of Korea

**Table S1.** Calculation of DIC score

| Variable | Value | Points |
| --- | --- | --- |
| Clinical symptoms and underlying diseases |  | 0 |
| Platelet count, ×10^3^/μL | < 50 | 0 |
|  | 50−100 | 1 |
|  | > 100 | 2 |
| D-dimer | No increase | 0 |
|  | Moderate increase | 2 |
|  | Strong increase | 3 |
| Fibrinogen, mg/dL | ≥ 100 | 0 |
|  | < 100 | 1 |
| Prothrombin time | < 3 sec | 0 |
|  | 3−6 sec | 1 |
|  | > 6 sec | 2 |
| Diagnosis of DIC |  | ≥ 5 |

**Table S2.** Clinical outcomes according to DIC by ECMO type

|  | **DIC** | **Non-DIC** | ***p*-value** |
| --- | --- | --- | --- |
| Venoarterial ECMO | (N=133) | (N=404) |  |
| In-hospital mortality | 69 (51.9) | 136 (33.7) | <0.001 |
| Bleeding | 14 (10.5) | 30 (7.4) | 0.343 |
| Thrombosis | 12 (9.0) | 22 (5.5) | 0.206 |
|  |  |  |  |
| Venovenous ECMO | (N=36) | (N=130) |  |
| In-hospital mortality | 24 (66.7) | 59 (45.4) | 0.038 |
| Bleeding | 6 (16.7) | 12 (9.2) | 0.334 |
| Thrombosis | 2 (5.6) | 7 (5.4) | >0.99 |
|  |  |  |  |
| Concomitant cardiac arrest | (N=78) | (N=213) |  |
| In-hospital mortality | 47 (60.3) | 95 (44.6) | 0.026 |
| Bleeding | 10 (12.8) | 15 (7.0) | 0.186 |
| Thrombosis | 10 (12.8) | 10 (4.7) | 0.030 |

Values are the n (%).

*DIC* disseminated intravascular coagulation, *ECMO* extracorporeal membrane oxygenation.

**Figure S1.** Clinical outcomes based on AT Ⅲ deficiency.


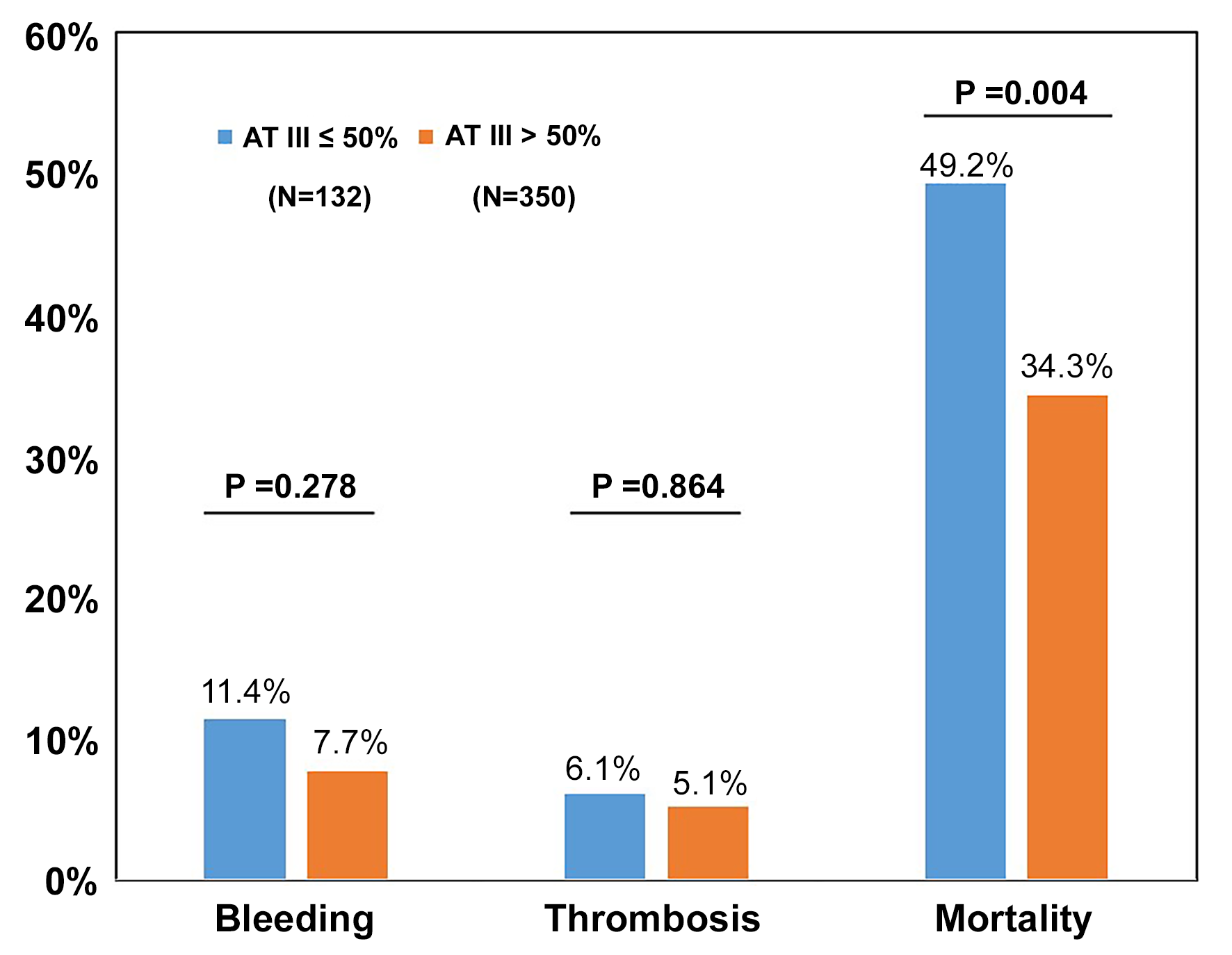


*AT* antithrombin, *DIC* disseminated intravascular coagulation.

**Figure S2.** The AUROC curve of AT Ⅲ for predicting overt DIC.





*AUROC* area under the receiver operating characteristic, *AT* antithrombin, *DIC* disseminated intravascular coagulation.
